# A Multicentric T-Cell Lymphoma with a Plasmacytoid Morphology in a Dog

**DOI:** 10.3390/vetsci5010010

**Published:** 2018-01-20

**Authors:** Alissa Bally, Karelma Frontera Acevedo, Indira Pargass, Lana Gyan, Stacy Rajh, Rod Suepaul

**Affiliations:** 1 The School of Veterinary Medicine, University of the West Indies, Saint Augustine, Trinidad and Tobago; Alissa.Bally@sta.uwi.edu (A.B.); Karelma.FronteraAcevedo@sta.uwi.edu (K.F.A.); Indira.Pargass@sta.uwi.edu (I.P.); stacyrajh@gmail.com (S.R.); 2 Veterinary Diagnostic Laboratory, Ministry of Agriculture, Land and Fisheries, Eric Williams Medical Sciences Complex, Uriah Butler Highway, Mt Hope, Trinidad and Tobago; lanaaditagyan@gmail.com

**Keywords:** plasmacytoid morphology, T-cell lymphoma, canine, pathology, immunohistochemistry, multicentric

## Abstract

An 8-year-old male (neutered) Labrador with a history of erythematous skin lesions and exercise intolerance for a prolonged period was suddenly found dead. Necropsy findings revealed an infiltrative, focally extensive mass which occupied 25% of the cardiac interventricular septum. Severe endocardiosis was also found on the bicuspid and tricuspid valves. The submandibular lymph nodes and kidneys were bilaterally enlarged, and the pre-hepatic lymph node and spleen were also enlarged. Multiple dermal pustules were present around the mouth and on the ear, and small ulcers were present on the tongue. Histopathological examination detected the presence of neoplastic lymphocytes with a plasmacytoid morphology in these tissues as well as in the tongue and skin lesions. Immunohistochemical (CD3^+^/CD18^+^) evaluation was consistent with a T-cell lymphoma, which could be classified as a peripheral T-cell lymphoma, not otherwise specified (PTCL-NOS).

## 1. Introduction

Approximately 80% of all hematopoietic tumors in dogs are lymphomas, which develop from neoplastic transformation of lymphocytes and commonly occur in lymph nodes. Lymphomas uncommonly arise from lymphocytes located in lymphoid centers outside lymph nodes including the gastrointestinal tract, spleen, tonsils, thymus, skin, nasal, and other tissues [1]. From an epidemiological perspective, the mean age of dogs suffering from lymphomas is 8 years, and Boxers have a higher incidence rate of lymphomas, particularly T-cell lymphomas [2]. Cytology and histopathology are preliminary tests conducted to confirm the presence of lymphoma, and immunophenotyping is used to determine if the cells are of B- or T-cell lineage. Approximately 30-40% of canine lymphomas are T-cell lymphomas [1].

The Revised European-American Lymphoma, World Health Organization (REAL/WHO) classification is the currently accepted scheme applied to lymphoma in dogs. This classification method uses morphology, immunophenotype, molecular characteristics, and biological behaviour [1]. The classification of lymphoma however, requires constant revision as each lymphoma may appear with a variety of morphological features and a range of clinical behaviour. [3]. Lymphomas with a plasmacytoid morphology are usually of B-cell origin and include plasmacytomas, lymphoplasmocytic lymphoma, plasmablastic lymphoma, myeloma, myeloid-related disorders, and osteosarcomas [1]. The term plasmacytoid T-cell lymphoma has been used for human lymphoma in the past; however, these tumors were later found to not be lymphomas [2,4,5].

Immunohistochemical confirmation of lymphomas is done by using the CD3 leukocyte antigen for T lymphocytes, and the CD79a leukocyte antigen for B lymphocytes and plasma cells [6]. Pax-5 and CD20 are commonly used in dogs as CD79a can cause artificial nuclear binding [3]. MUM1 is very specific for canine plasmacytomas with superior sensitivity and specificity compared to CD79a and CD 20 [7]. Although CD18 is utilized as a marker for histiocytic cells, it is present on the surface of all leukocytes and will stain some lymphocyte subtypes [8].

This report describes a case of a multicentric, CD3^+^/CD18^+^, T-cell lymphoma with a plasmacytoid morphology, which was diagnosed based on the histological characteristics of the cells and CD3^+^ positivity. To the best of the authors’ knowledge, this is the first report of this tumor type in the Caribbean.

## 2. Materials and Methods

A post mortem was conducted and grossly visible lesions were photographed, described, and identified. The tissues were fixed in 10% buffered formalin, processed routinely, and stained with hematoxylin and eosin (H&E).

Unstained sections on charged slides were shipped to the Pathology Diagnostic Laboratory, College of Veterinary Medicine, University of Georgia, where they were stained with immunohistochemical markers for CD79a (Dako, Carpinteria, CA, USA), MUM1 (Dako, Carpinteria, CA, USA), CD3 (Dako, Carpinteria, CA, USA) and CD18 (Dr. Peter Moore, Davis, CA, USA). Briefly, the slides were deparaffinized and underwent antigen retrieval, endogenous peroxidase blocking, and power block. Next, they were incubated with the primary antibodies at the following dilutions: CD3—1:1000, CD79a—1:1000, CD18—1:50, and MUM1—1:100. This incubation was followed by an incubation with the respective secondary antibodies: biotinylated anti-rabbit for CD3 (Vector Lab, Burlingame, CA, USA) and biotinylated anti-mouse for the rest (Vector Lab, Burlingame, CA, USA). Then they were incubated with streptavidin and horseradish peroxidase, followed by chromogen development using 3,3′-diaminobenzidine (DAB) and hematoxylin counterstaining. All of these used canine lymph nodes for a positive control and isotype control serum for a negative control. The IHC slides were evaluated by two anatomical pathologists using light microscopy for the determination of immunophenotype based on the staining of over 10% of the cells and the distribution of cells positive for CD3, MUM1, and CD79a.

## 3. Results

### 3.1. Animal Details

An 8-year-old male (neutered) Labrador was presented to the Veterinary Teaching Hospital (VTH), the School of Veterinary Medicine (SVM), University of the West Indies (UWI), Trinidad and Tobago for examination and assessment of pre-existing conditions which consisted of pruritic and erythematous skin and exercise intolerance. The dog was a member of the protective services, and for three weeks prior to presentation, he had been put off duty and treated by the attending veterinarian with cefuroxime at 10 mg/kg PO (oral administration), bid (twice daily) for 21 days, prednisolone at 1 mg/kg PO, sid (once daily) for 21 days, and ketoconazole weekly shampooing. The diet was also changed to a lamb and rice chow. On presentation to the VTH, the dog was bright, alert, and responsive. He had a body condition score (BCS) of 5/9, was panting at rest, and had a heart rate of 100 beats per minute (bpm) The pruritus and erythema had significantly decreased since the initial treatment three weeks prior, but the constant panting, likely related to the exercise intolerance, had not resolved. Due to the dog’s inability to adequately perform his duties, the clinician recommended that he be retired from duty with immediate effect.

A complete blood count (CBC), echocardiography (ECG), serum biochemistry, heartworm antigen test, and culture and sensitivity of a deep skin scrape were performed. The CBC, ECG, and the serum biochemistry detected no significant abnormalities. The heartworm antigen test was negative, and the culture and sensitivity test found *Staphylococcus intermedius*, and no yeasts or molds were detected. 

The handler was instructed to continue medical treatment for the dog’s skin condition using cefuroxime at 750 mgs sid for 14 days, followed by cefuroxime at 750 mgs sid every other day for 14 days. The oral prednisolone and ketoconazole shampooing were discontinued and the dog was to be maintained on a hypoallergenic lamb and rice chow diet. Two months after the dog’s last visit to the VTH, it developed an aural hematoma on the left pinna, which was successfully surgically treated. Three months after this, another aural hematoma developed on his right pinna which was also successfully surgically treated. The hematological and biochemical parameters were unremarkable both times and he appeared to be in good health. Seven months later, the animal was found dead and was brought to the SVM for necropsy.

### 3.2. Post Mortem Examination

The carcass was mildly autolyzed and the body condition of the animal was good (BCS 6/9), with adequate subcutaneous and internal fat deposits. The oral mucous membranes were pale pink. Four ulcers were present on the dorsal surface of the tongue, ranging from 2 to 5 mm in diameter. There were five small, 2–3 × 4–5 mm dermal pustules with surface crusting around the mouth and four similar lesions on each ear. The submandibular lymph nodes were bilaterally enlarged, ranging from 30–35 × 15–20 mm in size, and oozed serosanguinous fluid on cut surface. There were petechial hemorrhages scattered on the surface of the skin in the inguinal region. On the interventricular septum of the heart, there was an infiltrative, yellow-white to red (hemorrhagic), irregular, poorly circumscribed, firm, focally extensive mass, approximately 60 mm × 30 mm in size, which occupied approximately 20% of the myocardial tissue of the interventricular septum. On the bicuspid and tricuspid atrioventricular valves, there were multifocal cream, firm, smooth nodules, 2–5 mm in diameter, occupying 45% of the valvular leaflets. The kidneys were bilaterally firm, slightly pale, and enlarged (right: 25 × 100 mm and left 30 × 90 mm). The medullary regions of the kidney measured 15–25 mm in thickness from the pelvis to cortico-medullary junction. At the cranial pole of the right kidney, there was a pale to yellow/white, firm, sunken, irregular, focal lesion (likely due to chronic infarction) measuring 10 × 15 mm in size. The pre-hepatic lymph node was enlarged (50 × 30 mm in size), firm, and reddened and oozed serosanguinous fluid on cut surface.

The edges of the liver were slightly rounded and the lobes were diffusely dark red (congestion). Multiple petechial hemorrhages were distributed randomly throughout all lobes, occupying approximately 5% of the parenchyma. Throughout the pulmonary parenchyma, there were multifocal to coalescing patchy, irregular, dark red, regions. The spleen was slightly, diffusely enlarged and pale with dark red, multifocal (3–4 × 6–8 mm) to coalescing, well demarcated lesions on the capsular surface that extended into the splenic parenchyma. Stomach contents consisted of light brown, semi-solid ingesta. No other gross lesions were identified. Samples of the heart, kidneys, liver, lung, mandibular lymph node, spleen, brain, tongue and skin were placed in 10% neutral buffered formalin, embedded in paraffin, and 4-µm sections were taken for histopathological examination.

### 3.3. Histopathology

**Figure 1:**
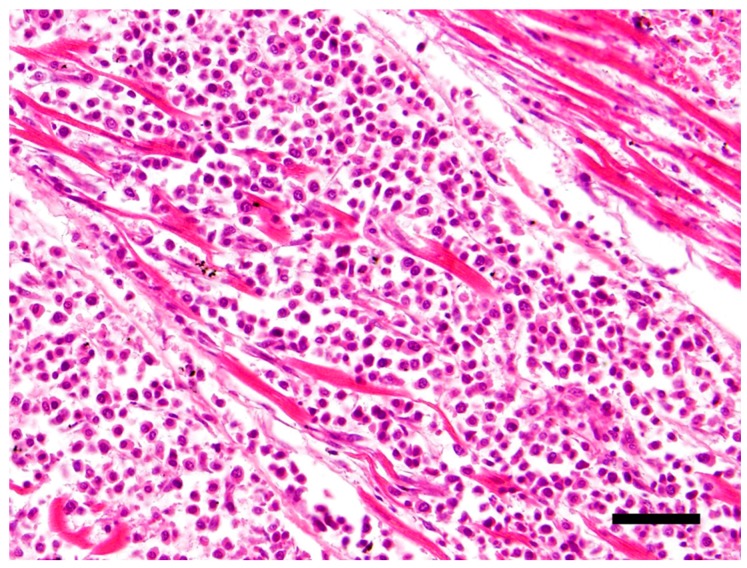
Effacement of the myocardial architecture by an invasive population of round cells that separate degenerating myofibers. Hematoxylin and eosin (H&E) stain 40×, Bar = 50μm.

**Figure 2:**
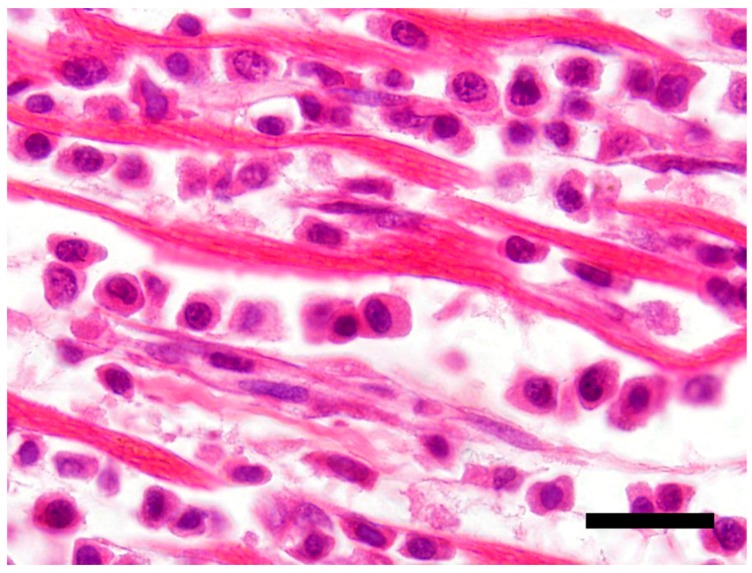
Neoplastic T-cells with a plasmacytoid appearance showing marked anisocytosis and anisokaryosis with moderate to large amounts of bright eosinophilic cytoplasm. H&E stain 100×, Bar = 20 μm.

The main histological finding was present in the heart. There was effacement of the myocardial architecture by an invasive population of round cells that separated degenerating myofibers (Figure 1). These neoplastic round cells showed marked anisocytosis and anisokaryosis, and had moderate to large amounts of bright eosinophilic cytoplasm and rare perinuclear clearing. The nuclei were round with speckled heterochromatin and mitoses averaged 4 per 10 hpf (high power (400x) fields). These neoplastic cells had a distinct plasmacytoid morphology (Figure 2).

The adjacent cardiac myocytes showed swelling, loss of striations and fragmentation. The atrioventricular valvular tissue was multifocally expanded by loose connective tissue (endocardiosis). Similar neoplastic cells were present throughout the kidneys in perivascular spaces, predominantly around middle-sized arterioles at the corticomedullary junction and extended into the medullary interstitium with the degeneration of adjacent tubules. There was swelling of tubular epithelial cells and eosinophilic thickening of the glomerular basement membranes. In the tongue, similar neoplastic cells were present in small to moderate numbers at multiple foci in the upper submucosa, with small numbers of these neoplastic cells invading the deeper epithelial layers of the mucosa of the tongue. Neoplastic cells were also present in a lymphatic vessel in the sub-mucosa of the tongue. Multiple small foci of neoplastic cells were present in the in the upper dermis of the skin, with small numbers of cells invading the epithelium. No lymphatic invasion was noted in the skin lesions. Similar neoplastic cells were present the sub-capsular and paracortical sinuses of the mandibular lymph node with the disruption of the cortical architecture, and the medullary areas were markedly congested and moderately autolyzed. The splenic tissue was autolyzed. There was congestion in the lungs and liver and no significant changes were detected in the brain.

### 3.4. Immunohistochemistry

**Figure 3:**
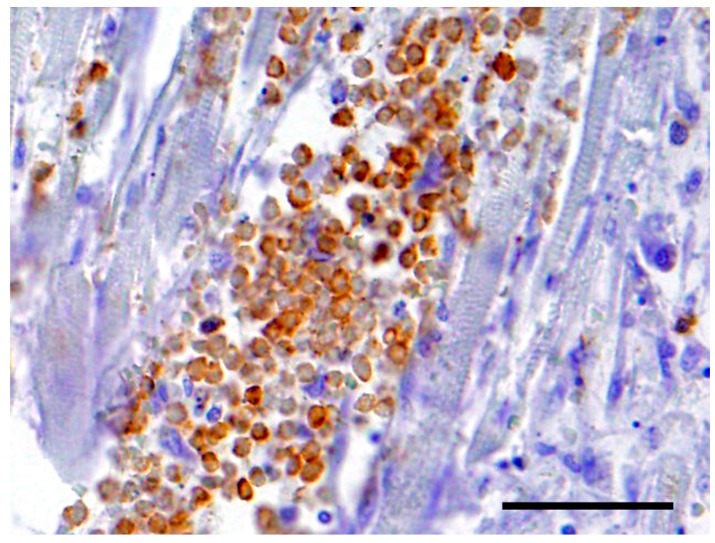
The neoplastic cells in the heart exhibited a diffuse, strong positive cytoplasmic staining for CD3^+^. Bar = 50 μm.

**Figure 4:**
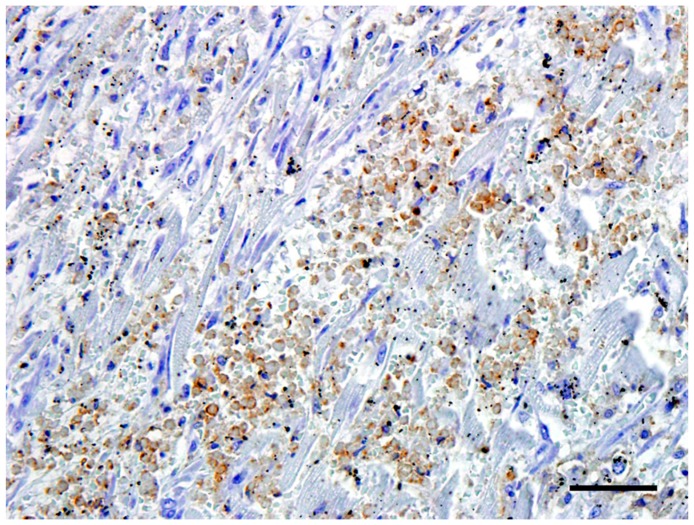
The neoplastic cells in the heart exhibited a mild to marked diffuse cytoplasmic staining for CD18. Bar = 50 μm.

The neoplastic cells in the heart exhibited a diffuse, strong positive membrane staining for CD3^+^ (Figure 3), thus confirming the presence of neoplastic T-lymphocytes in the heart. There was also a mild to marked diffuse membrane staining for CD18 (Figure 4). The cells showed no staining with CD79a and MUM1.

## 4. Discussion

The neoplastic cells displayed a distinct plasmacytoid morphology on routine H&E staining, and immunohistochemical staining was performed for confirmation of the cell type. However, the cells did not stain for CD79a and MUM1, IHC markers that are routinely used to confirm the presence of plasma cells [6,7]. They were positive for the T lymphocyte marker CD3, suggesting that the cells were T lymphocytes [6,8]. The epitheliotropism detected in the tongue and skin also support a T-cell lineage for these neoplastic cells. The term plasmacytoid was used to describe the morphology of the neoplastic T-cells (abundant, highly acidophilic cytoplasm, pale perinuclear area, and eccentric round nuclei). 

Application of the REAL/WHO classification system would most likely place this lymphoma in the category of a peripheral T-cell lymphoma, not otherwise specified (PTCL-NOS) [1], based on the morphological, immunohistochemical, and clinical characteristics. Given the epitheliotropic nature of the oral and skin lesions and the long-standing skin issues with this dog, it is also possible to place it in the category of cutaneous T-cell lymphoma (CTCL) with visceral spread [1]. While both these types of T-cell lymphoma are not rare, the plasmacytoid morphology of the neoplastic cells in this case is unusual, and differs from the typical cellular characteristics of PTCL-NOS and CTCL described in the literature. PTCL-NOS typically consists of intermediate to large cells with nuclei having prominent indentations, convolutions, or multilobulation, whilst in CTCL, intermediate-sized cells with round hyperchromatic and convoluted nuclei which occasionally have sharp shallow indentations are characteristically seen [3]. However, the morphological variation of lymphomas is wide and the remaining criteria for classification would allow classification into one of these two categories.

There is scant literature on T-cell lymphomas with plasmacytoid features, indicating that this is a rare type of T-cell lymphoma in which the morphology of the cells, especially in this case, could easily be mistaken for a plasma cell neoplasm. A gastric secretory plasmacytoma has recently been shown to also demonstrate CD3 positivity [9], however, the cells in our case did not stain with the typical CD79a and MUM1 markers. In this regard, PCR for Antigen Receptor Rearrangement (PARR) would have been a useful diagnostic adjunct to assist in determining the exact nature of the cells in this neoplasm.

The lymphoma in this dog had a multicentric distribution involving several tissues including the skin, tongue, kidneys, lymph nodes, and heart. The exact origin of the neoplasm could not be determined, thus affecting classification as either a CTCL or PTCL-NOS. CTCL lymphomas arise in the skin and can spread to the viscera [3]. However, given the morphological findings, the authors believe PTCL-NOS is the more applicable classification. It may also be possible that the heart was the primary site given the relative size of the mass detected in this organ and the long-standing exercise intolerance. 

## 5. Conclusions

Based on the pathological findings, histopathology and an immunophenotype of CD3^+^ and CD18^+^, a diagnosis of a PTCL-NOS with a plasmacytoid morphology was most likely. The plasmacytiod morphology noted in this case highlights the variability that can occur with lymphoma and the likely need for further immunophenotypical investigation into cases of lymphoma. In this case, the cardiac neoplasia, which resulted in the loss of a significant amount of cardiac musculature, along with the loss of valvular integrity associated with the severe endocardiosis, would likely have contributed to the exercise intolerance noted clinically. The chronic skin lesions were also associated with lymphoma.
